# The association between atypical adenomatous hyperplasia and primary lung cancer

**DOI:** 10.1054/bjoc.2000.1317

**Published:** 2000-08-16

**Authors:** A D Chapman, K M Kerr

**Affiliations:** Department of Pathology, Aberdeen Royal Infirmary and Aberdeen University Medical School, Foresterhill, Aberdeen, AB25 2ZD, UK

**Keywords:** AAH, adenocarcinoma, lung, preneoplasia, cancer screening

## Abstract

Atypical adenomatous hyperplasia (AAH) has been suggested as the adenoma in an adenoma–carcinoma sequence in the lung periphery. From 1989–1998, we undertook a systematic, prospective search for AAH in lungs resected for cancer. AAH was found in 67 of 554 patients (12.1%) with primary lung carcinoma (9.2% in male patients and 19.0% in females). AAH was found in lungs bearing adenocarcinoma (23.2%) more frequently than with large cell undifferentiated carcinoma (12.5%) or squamous carcinoma (3.3%). A greater percentage of females with adenocarcinoma had AAH (30.2%) than did males with adenocarcinoma (18.8%). Numbers of AAH ranged from 1–42 per patient and more patients had small numbers of AAH, although 12 patients had 6 or more AAH foci. Larger numbers of AAH tended to be found in adenocarcinoma-bearing lungs. Ten of the 67 patients with AAH and primary lung carcinoma (15%) had multiple primary cancers (range 2–6), all of which were adenocarcinoma. Synchronous cancers were rare in lung tumour-bearing resections without AAH. Patients with AAH show no difference in post-operative survival to those without, for all stages of carcinoma and for Stage I disease alone. This study provides evidence for a strong association between atypical adenomatous hyperplasia and primary lung adenocarcinoma and lends weight to the AAH/adenoma-carcinoma hypothesis. © 2000 Cancer Research Campaign


*British Journal of Cancer* (2000) **83**(5), 632–636
© 2000 Cancer Research Campaign
doi: 10.1054/ bjoc.2000.1317, available online at http://www.idealibrary.com on IDEAL®

### The association between atypical adenomatous hyperplasia and primary lung cancer

**AD Chapman and KM Kerr**

Department of Pathology, Aberdeen Royal Infirmary and Aberdeen University Medical School, Foresterhill, Aberdeen, AB25 2ZD, UK

**Summary** Atypical adenomatous hyperplasia (AAH) has been suggested as the adenoma in an adenoma–carcinoma sequence in the lung periphery. From 1989–1998, we undertook a systematic, prospective search for AAH in lungs resected for cancer. AAH was found in 67 of 554 patients (12.1%) with primary lung carcinoma (9.2% in male patients and 19.0% in females). AAH was found in lungs bearing adenocarcinoma (23.2%) more frequently than with large cell undifferentiated carcinoma (12.5%) or squamous carcinoma (3.3%). A greater percentage of females with adenocarcinoma had AAH (30.2%) than did males with adenocarcinoma (18.8%). Numbers of AAH ranged from 1–42 per patient and more patients had small numbers of AAH, although 12 patients had 6 or more AAH foci. Larger numbers of AAH tended to be found in adenocarcinoma-bearing lungs. Ten of the 67 patients with AAH and primary lung carcinoma (15%) had multiple primary cancers (range 2–6), all of which were adenocarcinoma. Synchronous cancers were rare in lung tumour-bearing resections without AAH. Patients with AAH show no difference in post-operative survival to those without, for all stages of carcinoma and for Stage I disease alone. This study provides evidence for a strong association between atypical adenomatous hyperplasia and primary lung adenocarcinoma and lends weight to the AAH/adenoma-carcinoma hypothesis. © 2000 Cancer Research Campaign

**Keywords:** AAH; adenocarcinoma; lung; preneoplasia; cancer screening

Atypical adenomatous hyperplazia (AAH) is a localised proliferation of alveolar lining cells, found particularly in the centriacinar regions of the peripheral alveolated lung. It has recently been a focus of attention after Miller et al (1988) suggested this lesion may be the adenoma in an adenoma–carcinoma sequence in the lung periphery, leading to the development of adenocarcinoma. This was based largely on the association of AAH lesions with peripheral type adenocarcinoma of the lung, a tumour classified as bronchioloalveolar carcinoma (BAC) and invasive adenocarcinoma with bronchioloalveolar features in the new WHO lung tumour classification (Travis et al, 1999). As well as the morphological similarity between large atypical AAH lesions and BAC, epidemiological data (Miller, 1990), morphometric and flow cytometric DNA analysis (Kodama et al, 1986; Nakayama et al, 1990; Mori et al, 1993), and molecular biological evidence particularly, including abnormal oncogene and tumour suppressor gene expression (Kerr et al, 1994; Hayashi et al, 1997; Kurasono et al, 1998), in addition to evidence of K-ras mutation (Westra et al, 1996; Cooper et al, 1997), have provided evidence to support this hypothesis. Recent data have shown that AAH lesions are clonal (Niho et al, 1999).

The identification of a potential precursor lesion for peripheral adenocarcinoma is of particular interest since the origin of this type of tumour was previously unknown, unlike the well known origin of central bronchogenic squamous carcinoma from bronchial squamous dysplasia/carcinoma-in-situ. Furthermore, there is evidence that adenocarcinoma is increasing in incidence in the Western world while squamous carcinoma incidence may be falling (Travis et al, 1995). These findings in the USA have been mirrored in the UK (Connolly et al, 1997) and in our own studies in NE Scotland (Kerr et al, 1996). Scotland has one of the highest lung cancer rates, in both males and females, in the world (Sharp and Brewster, 1999). Adenocarcinoma has long been the commonest form of lung cancer in Japan and in other Oriental populations (Shimosato et al, 1982) and it is notable that almost all of the literature on the subject of AAH concerns Japanese patients.

In this paper we present data on the association of AAH with lung cancer from a Scottish population and examine the influence of the presence of AAH on patient survival.

### MATERIALS AND METHODS

From 1989 to 1998 inclusive, we undertook a systematic prospective search for AAH in pulmonary resection specimens received in our department. Lung specimens were inflated perbronchially with 4% neutral buffered formal saline and allowed to fix in a reservoir of formalin for at least 24 hours. The specimens were sliced parasagittally at 1 cm thick intervals and carefully examined for AAH lesions, in addition to assessment of the main lesion for which the resection was performed. AAH lesions are often difficult to identify macroscopically due to their small size but can sometimes be identified as small yellow or grey foci, a few millimetres in diameter, on the cut surface of the lung. More often, however, they are found by chance on random sampling of the lung parenchyma, so that all foci suggestive of AAH were sampled and/or usually 1–6 random blocks of lung parenchyma were taken and examined histologically. The typical histological appearances of AAH are shown in Figure 1 with, for comparison, a BAC and invasive adenocarcinoma. AAH is characterized by alveoli lined by an often intermittent layer of cuboidal or low columnar cells

*Received 2 February 2000*
*Revised 28 April 2000*
*Accepted 8 May 2000*

*Correspondence to:* KM Kerr

A

B

C

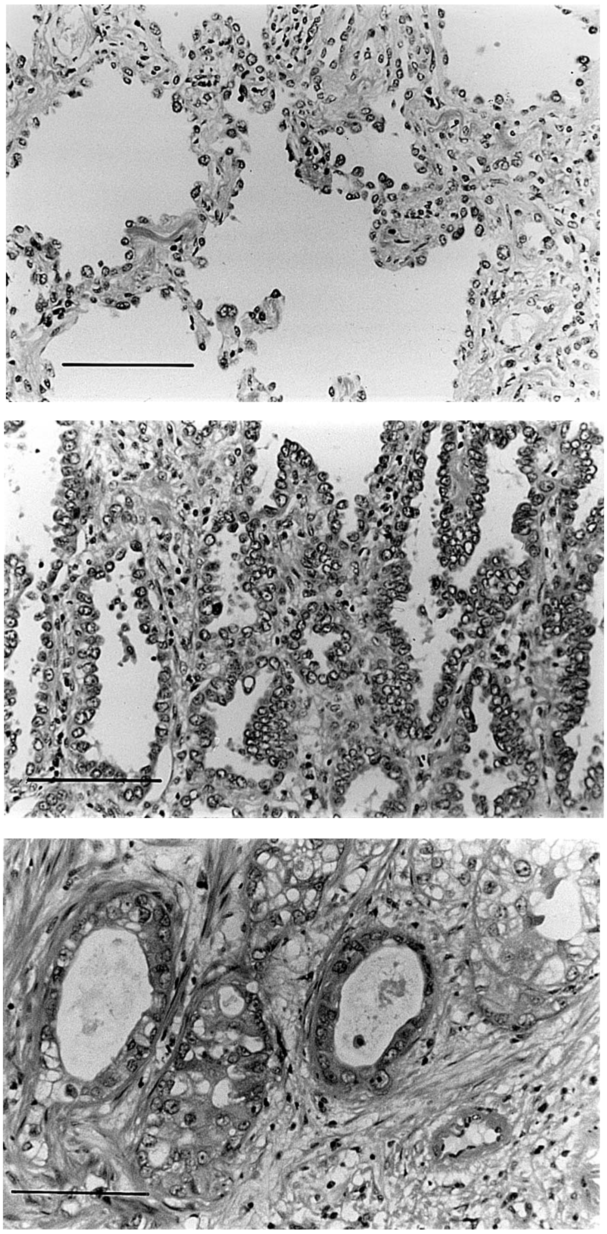

**Figure 1** (H&E, ×100). (**A**) Low grade atypical adenomatous hyperplasia. (**B**) Bronchioloalveolar carcinoma. Tumour cells line alveolar walls but there is no evidence of stromal invasion. (**C**) Invasive adenocarcinoma. Bar represents 200 μm

(Type 2 pneumocytes or Clara cells) exhibiting mild atypia. Mitoses are very rare and the alveolar walls are slightly thickened by variable amounts of collagen and fibroblasts. As AAH becomes more cellular and atypical, distinction from bronchioloalveolar carcinoma (a non-invasive lesion by the WHO definition) (Travis et al, 1999) may be difficult but the latter diagnosis is used in cases of larger lesions, usually over 1 cm where there is a complete lining of all the alveoli by a compact columnar cell population showing more atypia, close cell–cell apposition and a sharp distinction between the lesion and surrounding lung. The cells in BAC comprise a more homogeneous population than that usually seen in AAH. Similar features help distinguish AAH from a possible intrapulmonary metastasis from the associated primary tumour. Here the degree of cellular pleomorphism and greater cell size is the rule in a metastasis. In practice this latter distinction is virtually never an issue. The age and sex of each patient with AAH was noted and compared with data on patients without AAH. The histological type of the main cancer, the number of AAH lesions in each resection specimen and the number and type of any additional tumours were recorded for each patient with AAH. The local Cancer Registry provided information on whether or not the patients remained alive on the study date in February 1999. This information was used to compare postoperative survival in lung cancer patients with and without AAH, using the Kaplan-Meier method.

### RESULTS

From a total of 582 patients, 70 were found to have AAH (12%) (in specimens comprising 42 lobectomies, 22 pneumonectomies and 6 segmental/wedge resections). The age range for the patients with AAH was 36–84 years (mean age 62 years), almost identical to that for the entire study group (mean age 63 years). From the whole group, 554 patients (391 males and 163 females) had primary carcinoma of lung and of these, 138 males (35.3%) and 86 females (52.7%) had adenocarcinoma. Of the patients with AAH, 37 were male and 33 were female. Sixty-seven of the 70 patients with AAH underwent lung resection for primary lung cancer. Three patients, two females and one male, each with a single AAH lesion, had surgery for other tumours (metastatic sarcoma, metastatic rectal carcinoma and solitary fibrous tumour of pleura). Thus AAH was found in 12.1% of lungs bearing primary lung cancer (9.2% in male patients and 19.0% in females).

Fifty-two of the 67 patients with primary lung cancer and AAH had surgery for primary adenocarcinoma. AAH was found in 23.2% of adenocarcinoma bearing lungs but less often in those with large cell or squamous cell carcinoma (12.5% and 3.3% respectively) (Table 1). AAH was present in 30.2% of females, but in only 18.8% of males with adenocarcinoma.

The number of AAH lesions found in each resection specimen ranged from 1–42 (Table 2). Two thirds of the cases had 2 or more lesions, while 17% had 6 or more. Four patients had large numbers–12, 19, 34 and 42 lesions respectively, per patient. All patients with 6 or more AAH lesions had adenocarcinoma, except one who had a mixed tumour that included adenocarcinoma.

Ten of the 67 patients with primary lung carcinoma had multiple (2–6) cancers (15%) (including the 3 patients with most AAH lesions) and in all cases, the main primary and synchronous tumours were adenocarcinoma, the additional cancers usually being small (T1) tumours (Table 3).

Despite an apparent trend to better survival in the AAH group, there was no statistical difference in post-operative survival between those 70 patients with AAH and the remaining 512 patients whose resection specimens showed no evidence of AAH ($P$ = 0.21, Figure 2). The findings were the same when only patients with Stage I tumours were considered.

### DISCUSSION

In this prospective study of 582 patients, the incidence of AAH in the 554 lungs resected for primary lung cancer is at least 12.1% and is particularly strongly associated with primary adenocarcinoma (23.2%). Also, patients with adenocarcinoma are more

© 2000 Cancer Research Campaign

**Table 1** Number of patients with AAH in relation to histological type of main tumour

| Histological type of main tumour | Number of patients ($n$ = 582) | Number of patients with AAH ($n$ = 70) (%) |
|---|---|---|
| Adenocarcinoma | 224 | 52 (23.2) |
| Large cell carcinoma | 24 | 3 (12.5) |
| Squamous cell carcinoma | 214 | 7 (3.3) |
| Other primary carcinomas | 92 | 5 (5.4) |
| Other primary malignancy | 8 | 0 |
| Metastatic disease | 15 | 2 (13.3) |
| Benign tumours | 5 | 1 (20) |

**Table 2** Number of AAH lesions per case and comparison with published studies

| | Total patients with AAH | 1 AAH per case | 2 lesions per case | 3–5 lesions per case | 6–8 lesions per case | ≥9 lesions per case |
|---|---|---|---|---|---|---|
| No. of patients (Current study) | 70 | 24 | 17 | 17 | 8 | 4 |
| No. of patients (Weng et al, 1992) | 27 | 15 | 5 | 5 | 1 | 1 |
| No. of patients (Nakanishi, 1990) | 15 | 9 | 1 | 4 | 1 | |
| No. of patients (Miller, 1990) | 28 | 14 | 4 | 5 | | 5 |

**Table 3** Patients with AAH and multiple primary adenocarcinomas

| Patient | Number of AAH | Number of cancers in specimen[a] |
|---|---|---|
| 1 | 42 | 6 |
| 2 | 34 | 3 |
| 3 | 19 | 3 |
| 4 | 8 | 4 |
| 5 | 5 | 2 |
| 6 | 2 | 2 |
| 7 | 1 | 3 |
| 8, 9, 10 | 1 | 2 |

[a] All of these tumours were adenocarcinomas, of varying types according to the WHO classification (Travis et al, 1999). Many were invasive well differentiated adenocarcinoma with bronchioloalveolar features, especially the small additional cancers, while some were poorly differentiated.

likely to have larger numbers of AAH lesions when compared to those with other cancer types.

Our similar numbers of males and females with AAH contrasts with our previous report on 10 AAH patients, 7 of whom were women (Carey et al, 1992). In two Japanese studies, 11 of 15 (Nakanishi, 1990) and 22 of 27 (Weng et al, 1992) patients, respectively, were men, probably reflecting the greater frequency of lung cancer in males. Our greater frequency of AAH in women (19.0% of lung cancer resections, 30.2% of those specifically with adenocarcinoma) when compared to men (9.2% and 18.8% respectively) is interesting and contrasts with the study by Weng et al (1992), where 20% of the male, but only 4.8% of the female lung cancer patients had AAH. Why Scottish women should show a greater tendency to develop AAH, when compared to Japanese women, is not clear. Comparisons are difficult between cultural groups. Even though adenocarcinoma is proportionately more common in females, women with adenocarcinoma still have more AAH than do men with with adenocarcinoma.

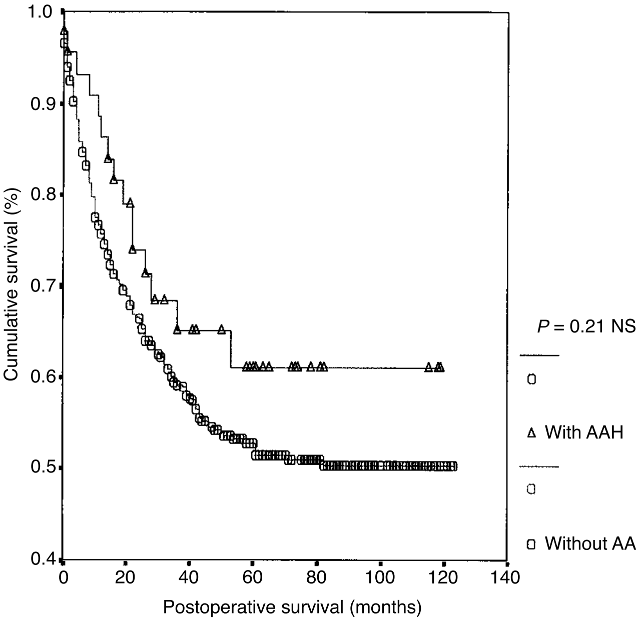

**Figure 2** Kaplan-Meier survival curves for patients with and without AAH. No significant difference exists between the two curves ($P$ = 0.21)

In the only other published non-Japanese study, from Vancouver, AAH was found in 9.3% of 247 lungs bearing a primary cancer (Miller, 1990). In four Japanese studies AAH was found in 12.2%, 13.9%, 16.4% and 21.4% of 131, 203, 210 and 70 resected lungs respectively (Morinaga and Shimosato, 1987; Kodama et al, 1988; Nakanishi, 1990; Weng et al, 1992). Noguchi and Shimosato (1995) reported AAH in 5.1% of 2098 cases but data were gathered from records spanning 1965–1989 and were not, apparently, the result of a prospective search for AAH. This

 © 2000 Cancer Research Campaign variation in published figures for AAH incidence is partly due to differences in sampling techniques used. It is clear that all studies will underestimate the number of lesions present and that the harder one looks, the more will be found.

AAH is less often found in lungs without a primary carcinoma. Two studies found AAH in, respectively, 4.4% and 9.6% of resections without primary carcinoma (Morinaga and Shimosato, 1987; Weng et al, 1992). We found AAH in 10.7% of such cases but our numbers are small. In a prospective autopsy study of 100 consecutive cases, only 2 patients were found to have AAH (Sterner et al, 1997). Neither had lung cancer. It is not clear what the incidence of AAH is in those without primary lung cancer, but it is lower than in those with the disease.

AAH is most common in lungs bearing adenocarcinoma. Our finding of AAH in 23.2% of such cases compares with 15.6%, 18.8%, 19.2%, 25.5% and 35.5%, respectively, in other series (Morinaga and Shimosato, 1987; Kodama et al, 1988; Miller, 1990; Nakanishi, 1990; Weng et al, 1992). The figures for squamous carcinoma bearing lungs are smaller: our figure of 3.3% is similar to the 3.0%, 5.9%, 6.9%, 9.4% and 11.1% of others (Morinaga and Shimosato, 1987; Kodama et al, 1988; Miller, 1990; Nakanishi, 1990; Weng et al, 1992). The relatively high frequency of AAH associated with large cell undifferentiated carcinoma is notable – 12.5% in our series versus 10% and 25% in the literature (Nakanishi, 1990; Weng et al, 1992) – particularly since there is some evidence from ultrastructural studies that most large cell undifferentiated carcinomas show adenocarcinomatous differentiation (Hammar, 1993). Central bronchial squamous carcinomas are more likely to show distal obstructive pneumonitis than peripheral adenocarcinomas, which will reduce the numbers of AAH found. Nonetheless, the association with adenocarcinoma does appear real.

AAH numbers per case vary. More patients have relatively few AAH lesions, a smaller number have several (Table 2). Other published studies concur with this trend but, like Miller (1990), we have a notable number of patients with many AAH foci. Miller's original proposal of this adenoma–carcinoma sequence came from the observation that adenocarcinoma is not infrequently multicentric and that larger numbers of AAH lesions are found in adenocarcinoma bearing lungs (Miller et al, 1988; Miller, 1990). Our ten patients with multiple adenocarcinomas associated with often large numbers of AAH lesions (Table 3) provide more evidence for the relationship between AAH and adenocarcinoma. Only 5 other patients are reported with multiple cancers, almost always adenocarcinoma, and multiple AAH (Nakanishi, 1990; Weng et al, 1990; Anami et al, 1998; Suzuki et al, 1998a). That 15% of our lung cancer patients with AAH have multiple synchronous primary tumours is interesting. Multiple primary malignancy is found in up to 2% of most reported series of resected tumours (Carey et al, 1993), including ours, when AAH cases are excluded. Two of our patients with AAH and adenocarcinoma had a second (metachronous) lung cancer, one of which was also adenocarcinoma. In all our cases where there were multiple adenocarcinomas, there was clear morphological evidence that these were not metastases from the main lesion. We found no difference in post-operative survival between those patients with and without AAH, both overall and for Stage I disease only. This is a similar finding to the work of Suzuki et al (1997). Takigawa et al (1999), however, suggested that patients with stage IA adenocarcinoma and AAH may have a better prognosis than those with the same cancer but no AAH, though this difference did not reach statistical significance.

This study provides evidence of a strong association between AAH and adenocarcinoma in the lung in a Western Caucasian population, similar to the experience in Japan. These data underpin the proposed AAH/adenoma–carcinoma sequence in the lung, a concept further strengthened by various immunohistochemical and molecular biological studies (Kitamura et al, 1999; Kerr, 2000). The finding of multiple AAH lesions in patients with multiple lung cancers, together with the report of AAH in a patient with multiple synchronous lung cancers in Li-Fraumeni syndrome (Nadav et al, 1998) and multiple AAH lesions associated with tumours which have deletions in the tuberous sclerosis complex (TSC-1) region on 9 q (Suzuki et al, 1998b), raises interesting questions about possible underlying genetic abnormalities in this disease. The idea that multiple AAH may represent a form of 'field cancerization' in the lung periphery is also an attractive one, similar to the widespread genotypic changes which parallel the multifocal phenotypic changes seen in the bronchial epithelium in many tobacco smokers (Wistuba et al, 1999). Niho et al (1999) have demonstrated monoclonality in AAH with clonal heterogeneity between multiple AAH lesions from the same patient. This multifocal 'field' effect is supported by some similar studies on BAC (Barsky et al, 1994), but not by others (Holst et al, 1998). The survival studies are an inadequate tool to determine much about the natural history of AAH lesions since patient outcome will be determined to a greater extent by the intercurrent cancer present in almost all patients. Until we have more patients with AAH, but without cancer, for extended follow-up or are even able to observe AAH longitudinally in a patient over a long time period, detail of the natural history of AAH will remain elusive. The rising incidence of primary lung adenocarcinoma in Western countries, together with the increase in lung cancer in women (who develop more adenocarcinoma than men and who seem, at least in Scotland, to be more likely to develop AAH) highlight the need for greater awareness of the precursor of this disease. This is particularly true if any progress is to be made with screening for this common fatal malignancy.

### ACKNOWLEDGEMENTS

We are grateful to Mr JS Cockburn and Mr RR Jeffrey, Department of Cardiothoracic Surgery, Aberdeen Royal Infirmary, who performed most of the pulmonary resections on patients in our study.

### REFERENCES

Anami Y, Matsuno Y, Yamada T, Takeuchi T, Nakayama H, Hirohashi S and Noguchi M (1998) A case of double primary adenocarcinoma of the lung with multiple atypical adenomatous hyperplasia. *Pathol Int* **48**: 634–640

Barsky SH, Grossman DA, Ho J and Holmes EC (1994) The multifocality of bronchioloalveolar lung carcinoma: Evidence and implications of a multiclonal origin. *Mod Pathol* **7**: 633–640

Carey FA, Wallace WAH, Fergusson RJ, Kerr KM and Lamb D (1992) Alveolar atypical hyperplasia in association with primary pulmonary adenocarcinoma: a clinicopathological study of 10 cases. *Thorax* **47**: 1041–1043

Carey FA, Donnelly SC, Walker WS, Cameron EW and Lamb D (1993) Synchronous primary lung cancers: prevalence in surgical material and clinical implications. *Thorax* **48**: 344–346

Connolly CK, Crawford SM, Rider PL, Smith AD, Johnston CF and Muers MF (1997) Carcinoma of the bronchus in the Yorkshire region of England 1976–1990: trends since 1984. *Eur Resp J* **10**: 397–403

*© 2000 Cancer Research Campaign*

Cooper CA, Carey FA, Bubb VJ, Lamb D, Kerr KM and Wyllie AH (1997) The pattern of K-ras mutation in pulmonary adenocarcinoma defines a new pathway of tumour development in the human lung. *J Pathol* **181**: 401–404

Hammar SP (1993) Common neoplasms. In: *Pulmonary Pathology*, Dail DH, Hammar SP (eds) pp 1123–1278. Springer-Verlag: New York

Hayashi H, Miyamoto H, Ito T, Kameda Y, Nakamura N, Kubota Y and, Kitamura H (1997) Analysis of p21Waf1/Cip1 expression in normal, premalignant, and malignant cells during the development of human lung adenocarcinoma. *Am J Pathol* **151**: 461–470

Holst VA, Finkelstein S and Yousem SA (1998) Bronchioloalveolar adenocarcinoma of lung. Monoclonal origin for multifocal disease. *Am J Surg Pathol* **22**: 1343–1350

Kerr KM (2000) Pulmonary preinvasive neoplasia. *J Clin Pathol* (in press)

Kerr KM, Carey FA, King G and Lamb D (1994) Atypical alveolar hyperplasia: relationship with pulmonary adenocarcinoma, p53, and c-erbB-2 expression. *J Pathol* **174**: 249–256

Kerr KM, Johnson SK, Kennedy MM and Jeffrey RR (1996) Rising incidence of primary lung adenocarcinoma in North East Scotland. *Thorax* **51**: A56

Kitamura H, Kameda Y, Ito T and Hayashi H (1999) Atypical adenomatous hyperplasia of the lung. Implications for the pathogenesis of peripheral lung adenocarcinoma. *Am J Clin Pathol* **111**: 610–622

Kodama T, Biyajima S, Watanabe S and Shimosato Y (1986) Morphometric study of adenocarcinoma and hyperplastic epithelial lesions in the peripheral lung. *Am J Clin Pathol* **85**: 146–151

Kodama T, Nishiyama H, Nishiwaki Y, Takanashi K, Kuroki M, Hayashibe A, Nukariya N, Kitaya T and Matusuyama T (1988) Histopathological study of adenocarcinoma and hyperplastic epithelial lesions of the Lung. *Lung Cancer (Haigan)* **8**: 325–333

Kurasono Y, Ito T, Kameda Y, Nakamura N and Kitamura H (1998) Expression of cyclin D1, retinoblastoma gene protein, and p16 MTS1 protein in atypical adenomatous hyperplasia and adenocarcinoma of the lung. An immunohistochemical analysis. *Virchows Arch* **432**: 207–215

Miller RR (1990) Bronchioloalveolar cell adenomas. *Am J Surg Pathol* **14**: 904–912

Miller RR, Nelems B, Evans KG, Müller NL and Ostrow DN (1988) Glandular neoplasia of the lung. A proposed analogy to colonic tumours. *Cancer* **61**: 1009–1014

Mori M, Chiba R and Takahashi T (1993) Atypical adenomatous hyperplasia of the lung and its differentiation from adenocarcinoma. *Cancer* **72**: 2331–2340

Morinaga S and Shimosato Y (1987) Pathology of the microadenocarcinoma in the periphery of the lung. *Pathol Clin Med Jpn* **5**: 74–80

Nadav Y, Pastorino U and Nicholson AG (1998) Multiple synchronous lung cancers and atypical adenomatous hyperplasia in Li-Fraumeni syndrome. *Histopathol* **33**: 52–54

Nakanishi K (1990) Alveolar epithelial hyperplasia and adenocarcinoma of the lung. *Arch Pathol Lab Med* **114**: 363–368

Nakayama H, Noguchi M, Tsuchiya R, Kodama T and Shimosato Y (1990) Clonal growth of atypical adenomatous hyperplasia of the lung: cytofluorometric analysis of nuclear DNA content. *Mod Pathol* **3**: 314–320

Niho S, Yokose T, Suzuki K, Kodama T, Nishiwaki Y and Mukai K (1999) Monoclonality of atypical adenomatous hyperplasia of the lung. *Am J Pathol* **154**: 249–254

Noguchi M and Shimosato Y (1995) The development and progression of adenocarcinoma of the lung. *Cancer Treat Res* **72**: 131–142

Sharp L, Brewster D (1999) The epidemiology of lung cancer in Scotland: a review of trends in incidence, survival and mortality and prospects for prevention. *Health Bull* **57**: 318–331

Shimosato Y, Kodama T and Kameya T (1982) Morphogenesis of peripheral type adenocarcinoma of the lung. In: *Morphogenesis of lung cancer Volume 1*, Shimosato Y, Melamed MR, Nettesheim P (eds) pp 65–90. CRC Press: Boca Raton, FI

Sterner DJ, Mori M, Roggli VL and Fraire AE (1997) Prevalence of pulmonary atypical alveolar cell hyperplasia in an autopsy population: a study of 100 cases. *Mod Pathol* **10**: 469–473

Suzuki K, Nagai K, Yoshida J, Yokose T, Kodama T, Takahashi K, Nishimura M, Kawasaki H, Yokozaki M and Nishiwaki Y (1997) The prognosis of resected lung carcinoma associated with atypical adenomatous hyperplasia: a comparison of the prognosis of well-differentiated adenocarcinoma associated with atypical adenomatous hyperplasia and intrapulmonary metastasis. *Cancer* **79**: 1521–1526

Suzuki K, Takahashi K, Yoshida J, Nishimura M, Yokose T, Nishiwaki Y and Nagai K (1998a) Synchronous double primary lung carcinomas associated with multiple atypical adenomatous hyperplasia. *Lung Cancer* **19**: 131–139

Suzuki K, Ogura T, Yokose T, Nagai K, Mukai K, Kodama T, Nishiwaki Y and Esumi H. (1998b) Loss of heterozygosity in the tuberous sclerosis gene associated regions in adenocarcinoma of the lung accompanied by multiple atypical adenomatous hyperplasia. *Int J Cancer (Pred Oncol)* **79**: 384–389

Takigawa N, Segawa Y, Nakata M, Saeki H, Mandai K, Kishino D, Shimono M, Ida M and Eguchi K (1999) Clinical investigation of atypical adenomatous hyperplasia of the lung. *Lung Cancer* **25**: 115–121

Travis WD, Travis LB and Devesa SS (1995) Lung Cancer. *Cancer* **75(Suppl)**: 191–202

Travis WD, Colby TV, Corrin B, Shimosato Y and Brambilla E (1999) *Histological typing of lung and pleural tumours*. WHO International histological classification of tumours, 3rd edition. Springer: Berlin

Weng S, Tsuchiya E, Satoh Y, Kitagawa T, Nakagawa K and Sugano H (1990) Multiple atypical adenomatous hyperplasia of type II pneumocytes and bronchiolo-alveolar carcinoma. *Histopathol* **16**: 101–103

Weng SY, Tsuchiya E, Kasuga T and Sugano H (1992) Incidence of atypical bronchioloalveolar cell hyperplasia of the lung: relation to histological subtypes of lung cancer. *Virchows Arch* **420**: 463–471

Westra WH, Baas IO, Hruban RH, Askin FB, Wilson K, Offerhaus GJ and Slebos RJ (1996) K-ras oncogene activation in atypical alveolar hyperplasias of the human lung. *Cancer Res* **56**: 2224–2228

Wistuba II, Behrens C, Milchgrub S, Bryant D, Hung J, Minna JD and Gazdar AF (1999) Sequential molecular abnormalities are involved in the multistage development of squamous cell lung carcinoma. *Oncogene* **18**: 643–650

*© 2000 Cancer Research Campaign*
